# Supplementary material for: Allosteric regulation and crystallographic fragment screening of SARS-CoV-2 NSP15 endoribonuclease
Source: Nucleic Acids Res. 2023 Apr 28;51(10):5255–70. doi: 10.1093/nar/gkad314 (PMC10250223; doi:10.1093/nar/gkad314)
Supplement: gkad314_Supplemental_Files [file gkad314_supplemental_files.zip › Supplementary datasets.zip!/231/Refine_8/01-BUSTER/Cycle-4/shell.01/R_Rfree.html]

# Rxpct factors in resolution bins at cycle 0


|  |  |  |  |  |  |  |  |  |
| --- | --- | --- | --- | --- | --- | --- | --- | --- |
|  | **<d> (Å)**  Rxpct\_Work | Rxpct\_Free | Rxpct\_All | |  |  |  |  |
|  | 13.1576 | 0.3319 | 0.3914 | 0.3344 |  |
|  | 7.5965 | 0.2778 | 0.3067 | 0.2795 |  |
|  | 5.8843 | 0.2860 | 0.2939 | 0.2863 |  |
|  | 4.9731 | 0.2134 | 0.2517 | 0.2155 |  |
|  | 4.3859 | 0.1983 | 0.1965 | 0.1982 |  |
|  | 3.9672 | 0.2066 | 0.2259 | 0.2075 |  |
|  | 3.6493 | 0.2268 | 0.2674 | 0.2288 |  |
|  | 3.3973 | 0.2294 | 0.2614 | 0.2312 |  |
|  | 3.1912 | 0.2263 | 0.2271 | 0.2264 |  |
|  | 3.0186 | 0.2169 | 0.2262 | 0.2173 |  |
|  | 2.8712 | 0.2150 | 0.2154 | 0.2151 |  |
|  | 2.7435 | 0.2034 | 0.2274 | 0.2046 |  |
|  | 2.6315 | 0.1999 | 0.2251 | 0.2014 |  |
|  | 2.5322 | 0.1886 | 0.1885 | 0.1886 |  |
|  | 2.4433 | 0.1819 | 0.1906 | 0.1823 |  |
|  | 2.3632 | 0.1837 | 0.1878 | 0.1839 |  |
|  | 2.2904 | 0.1808 | 0.1751 | 0.1805 |  |
|  | 2.2240 | 0.1959 | 0.2073 | 0.1964 |  |
|  | 2.1631 | 0.1962 | 0.2170 | 0.1972 |  |
|  | 2.1069 | 0.2369 | 0.2277 | 0.2364 |  |
|  | **Overall** | 0.2247 | 0.2427 | 0.2256 |  |

# Rxpct factors in resolution bins at cycle 1


|  |  |  |  |  |  |  |  |  |
| --- | --- | --- | --- | --- | --- | --- | --- | --- |
|  | **<d> (Å)**  Rxpct\_Work | Rxpct\_Free | Rxpct\_All | |  |  |  |  |
|  | 13.1576 | 0.3323 | 0.3920 | 0.3348 |  |
|  | 7.5965 | 0.2780 | 0.3068 | 0.2797 |  |
|  | 5.8843 | 0.2859 | 0.2941 | 0.2862 |  |
|  | 4.9731 | 0.2131 | 0.2520 | 0.2153 |  |
|  | 4.3859 | 0.1979 | 0.1964 | 0.1979 |  |
|  | 3.9672 | 0.2061 | 0.2256 | 0.2070 |  |
|  | 3.6493 | 0.2260 | 0.2668 | 0.2280 |  |
|  | 3.3973 | 0.2284 | 0.2607 | 0.2302 |  |
|  | 3.1912 | 0.2251 | 0.2266 | 0.2252 |  |
|  | 3.0186 | 0.2157 | 0.2252 | 0.2162 |  |
|  | 2.8712 | 0.2140 | 0.2151 | 0.2140 |  |
|  | 2.7435 | 0.2024 | 0.2269 | 0.2036 |  |
|  | 2.6315 | 0.1993 | 0.2253 | 0.2008 |  |
|  | 2.5322 | 0.1883 | 0.1885 | 0.1883 |  |
|  | 2.4433 | 0.1820 | 0.1906 | 0.1823 |  |
|  | 2.3632 | 0.1839 | 0.1882 | 0.1841 |  |
|  | 2.2904 | 0.1809 | 0.1760 | 0.1807 |  |
|  | 2.2240 | 0.1959 | 0.2071 | 0.1964 |  |
|  | 2.1631 | 0.1964 | 0.2174 | 0.1974 |  |
|  | 2.1069 | 0.2371 | 0.2278 | 0.2366 |  |
|  | **Overall** | 0.2243 | 0.2426 | 0.2252 |  |

# Rxpct factors in resolution bins at cycle 2


|  |  |  |  |  |  |  |  |  |
| --- | --- | --- | --- | --- | --- | --- | --- | --- |
|  | **<d> (Å)**  Rxpct\_Work | Rxpct\_Free | Rxpct\_All | |  |  |  |  |
|  | 13.1576 | 0.3326 | 0.3924 | 0.3351 |  |
|  | 7.5965 | 0.2781 | 0.3067 | 0.2798 |  |
|  | 5.8843 | 0.2856 | 0.2940 | 0.2859 |  |
|  | 4.9731 | 0.2127 | 0.2522 | 0.2150 |  |
|  | 4.3859 | 0.1975 | 0.1963 | 0.1975 |  |
|  | 3.9672 | 0.2055 | 0.2254 | 0.2064 |  |
|  | 3.6493 | 0.2252 | 0.2663 | 0.2272 |  |
|  | 3.3973 | 0.2275 | 0.2601 | 0.2292 |  |
|  | 3.1912 | 0.2241 | 0.2262 | 0.2242 |  |
|  | 3.0186 | 0.2147 | 0.2247 | 0.2152 |  |
|  | 2.8712 | 0.2131 | 0.2148 | 0.2132 |  |
|  | 2.7435 | 0.2017 | 0.2263 | 0.2029 |  |
|  | 2.6315 | 0.1987 | 0.2256 | 0.2003 |  |
|  | 2.5322 | 0.1882 | 0.1886 | 0.1882 |  |
|  | 2.4433 | 0.1820 | 0.1908 | 0.1824 |  |
|  | 2.3632 | 0.1841 | 0.1886 | 0.1844 |  |
|  | 2.2904 | 0.1812 | 0.1763 | 0.1810 |  |
|  | 2.2240 | 0.1960 | 0.2076 | 0.1965 |  |
|  | 2.1631 | 0.1966 | 0.2176 | 0.1976 |  |
|  | 2.1069 | 0.2374 | 0.2279 | 0.2368 |  |
|  | **Overall** | 0.2238 | 0.2424 | 0.2248 |  |

# Rxpct factors in resolution bins at cycle 3


|  |  |  |  |  |  |  |  |  |
| --- | --- | --- | --- | --- | --- | --- | --- | --- |
|  | **<d> (Å)**  Rxpct\_Work | Rxpct\_Free | Rxpct\_All | |  |  |  |  |
|  | 13.1576 | 0.3354 | 0.4100 | 0.3385 |  |
|  | 7.5965 | 0.2779 | 0.3011 | 0.2793 |  |
|  | 5.8843 | 0.2671 | 0.2837 | 0.2678 |  |
|  | 4.9731 | 0.1987 | 0.2288 | 0.2004 |  |
|  | 4.3859 | 0.1824 | 0.1779 | 0.1822 |  |
|  | 3.9672 | 0.1888 | 0.2119 | 0.1899 |  |
|  | 3.6493 | 0.2065 | 0.2529 | 0.2088 |  |
|  | 3.3973 | 0.2060 | 0.2335 | 0.2075 |  |
|  | 3.1912 | 0.2055 | 0.2150 | 0.2060 |  |
|  | 3.0186 | 0.1969 | 0.2138 | 0.1976 |  |
|  | 2.8712 | 0.1967 | 0.2127 | 0.1975 |  |
|  | 2.7435 | 0.1875 | 0.2205 | 0.1891 |  |
|  | 2.6315 | 0.1894 | 0.2186 | 0.1911 |  |
|  | 2.5322 | 0.1839 | 0.1866 | 0.1840 |  |
|  | 2.4433 | 0.1831 | 0.1947 | 0.1836 |  |
|  | 2.3632 | 0.1886 | 0.1957 | 0.1890 |  |
|  | 2.2904 | 0.1862 | 0.1885 | 0.1863 |  |
|  | 2.2240 | 0.2014 | 0.2207 | 0.2022 |  |
|  | 2.1631 | 0.2034 | 0.2262 | 0.2045 |  |
|  | 2.1069 | 0.2437 | 0.2321 | 0.2430 |  |
|  | **Overall** | 0.2121 | 0.2326 | 0.2131 |  |
